# Mapping the hepatic regulatory landscape of estradiol in gonadectomized obese female and male mice at single-nucleus resolution

**DOI:** 10.1186/s13293-026-00962-1

**Published:** 2026-08-07

**Authors:** Yutian Zhao, Ruirui Lan, In Sook Ahn, Sophia Yu, Guanglin Zhang, Graciel Diamante, Bridget Litts, Ariel Thorson, Kelsey P. Schaefers, Xia Yang, John M. Stafford

**Affiliations:** 1 Department of Integrative Biology and Physiology, University of California, Los Angeles, Los Angeles, CA USA; 2 Molecular, Cellular, and Integrative Physiology Interdepartmental Ph.D. Program, University of California, Los Angeles, Los Angeles, CA USA; 3 Department of Medicine, Division of Diabetes, Endocrinology and Metabolism, Vanderbilt University Medical Center, Nashville, TN USA; 4 Department of Molecular Physiology & Biophysics, Vanderbilt University, Nashville, TN USA; 5 Department of Molecular and Medical Pharmacology, University of California, Los Angeles, Los Angeles, CA USA; 6 Department of Medicine, Division of Endocrinology, Diabetes, and Metabolism, The Ohio State University, Wexner Medical Center, Columbus, OH USA

**Keywords:** Estradiol, Estrogen therapy, Liver, Liver zonations, Single-nucleus RNA sequencing, Obesity, Sex difference

## Abstract

**Background:**

Obesity is a prevalent, systemic metabolic disease affecting not only various adipose depots but also the liver, where lipid accumulation results in metabolic dysfunction-associated fatty liver disease, dyslipidemia, and insulin resistance. While estradiol (E2) has been known to be hepatoprotective for glucose and lipid metabolism in females, whether E2’s effects on individual liver cell types are shared between sexes or are sex-specific with obesity remains poorly defined.

**Methods:**

We performed single-nucleus RNA sequencing on liver tissue of E2-treated obese, gonadectomized male (XY) and female (XX) mice with diet-induced obesity to evaluate the sex-concordant and discordant effects of chronic E2 treatment. Differential gene expression, pathway analysis, intracellular and intercellular regulatory network modeling, and human metabolic disease/trait association analysis were conducted.

**Results:**

E2-induced transcriptional effects among hepatocyte subtypes were largely sex-concordant in both males and females, along with sex-specific E2 changes. In pericentral hepatocytes, E2 concordantly reduced the expression of genes involved in insulin resistance, FOXO signaling, and lipid biosynthesis; in periportal hepatocytes, E2 treatment enhanced cholesterol efflux genes and suppressed oxidative stress response genes. XY-specific effects of E2 included additional suppression of lipid and energy metabolic pathways in pericentral hepatocytes and oxidative phosphorylation in periportal hepatocytes, while in XX mice E2 showed unique regulation of glucose and lipid utilization programs. Network analysis revealed that E2-responsive genes in hepatocyte subtypes in both sexes showed enriched transcription factors associated with estrogen receptor signaling and lipid sensing and metabolism, alongside sex-specific regulators. Intercellular signaling affected by E2 showed more changes in XY mice than in XX mice across hepatocyte subtypes. Lastly, while E2-responsive genes in hepatocyte subtypes of both sexes were enriched for human genetic signals associated with lipid profiles and coronary artery disease, we also identified sex-specific associations, such as the link of E2-downregulated genes in XY hepatocytes to body mass index in men.

**Conclusions:**

Our findings revealed that chronic E2 administration remodels gene regulatory programs in individual liver cell types through both shared and sex-specific mechanisms, highlighting the importance of considering sex chromosome-dependent E2 responses when developing therapeutic strategies for obesity and associated hepatic metabolic dysfunction.

**Supplementary Information:**

The online version contains supplementary material available at https://doi.org/10.1186/s13293-026-00962-1.

## Abstract (plain language summary)

Obesity is a growing global health concern that has negative impacts on a broad spectrum of organs, including the liver. Estradiol (E2) is a sex hormone that generally plays a protective role in both sexes against liver dysfunctions. However, whether and how E2 effects differ between sexes and across various liver cell types remains understudied. Here, we compared and contrasted how liver cells responded to chronic E2 treatment between obese female and male mice. We found that hepatocytes, the major liver cell type, generally responded to E2 in similar ways in both sexes. In hepatocytes located near the liver’s central vein, E2 dampened shared pathways related to insulin resistance and lipid synthesis in both sexes, while additionally suppressing lipid and energy metabolic processes in males only. In hepatocytes near the portal triad, where oxygenated and nutrient-rich blood arrives, E2 enhanced cholesterol processing and reduced oxidative stress response in both sexes. E2 also induced more cell–cell communication changes in male mice than in female mice across hepatocyte subtypes. Lastly, E2-responsive genes in hepatocyte subtypes of both sexes were largely associated with lipid profiles and heart disease, with additional sex-specific obesity and diabetes associations also observed.

**Supplementary Information:**

The online version contains supplementary material available at https://doi.org/10.1186/s13293-026-00962-1.

## Highlights

Single-nucleus RNA sequencing revealed sex-concordant and discordant genes and pathways in hepatic cell types of obese gonadectomized mice, with hepatocyte subtypes showing greater concordance between sexes.

Shared E2 effects include dampened insulin resistance, FOXO signaling, and lipid biosynthesis in pericentral hepatocytes, along with enhanced cholesterol efflux and suppressed oxidative stress response in periportal hepatocytes.

Sex-discordant effects were also observed: in XY mice, lipid and energy metabolic pathways were additionally suppressed in pericentral and periportal hepatocytes, while in XX mice, unique regulation of glucose and lipid utilization programs was observed.

Cell–cell communication analysis revealed more E2-induced signaling changes in XY mice than in XX mice across hepatocyte subtypes.

E2-responsive genes in hepatocytes of both sexes were enriched for human genetic signals associated with lipid profiles and heart diseases, while cell type- and sex-specific associations with obesity and diabetes were also observed.

**Supplementary Information:**

The online version contains supplementary material available at https://doi.org/10.1186/s13293-026-00962-1.

## Background

Obesity, characterized by excessive fat accumulation, has become a global pandemic. Obesity is associated with escalating prevalence of cardiovascular disease, diabetes, dyslipidemia, and metabolic dysfunction-associated steatotic liver disease (MASLD). Chronic high-fat diet (HFD)-induced obesity is common in patients with MASLD, the most prevalent chronic liver disease that progresses from steatosis to steatohepatitis, fibrosis, and cirrhosis [1–3].

Epidemiological studies have revealed marked sex disparities in MASLD pathophysiology [4–6]. Males typically develop higher hepatic fat deposition and earlier progression to advanced MASLD stages, whereas premenopausal females are relatively protected through estrogen-mediated mechanisms that diminish after menopause [7]. Notably, aspects of the protective metabolic effects of endogenous estrogens in females can be restored via hormone treatment after menopause [8–10]. Estrogens have a wide range of effects on metabolic and inflammatory pathways, especially in the liver, such as upregulating glucose metabolism, lipid metabolism, and liver regeneration and inhibiting JNK and NF-kB pathways, and the lack of estrogen promotes multisystem metabolic dysfunction that leads to MASLD development [11–14]. In males, low fasting serum estradiol (E2, the main form of estrogen in humans) levels have been associated with elevated risk of cardiovascular disease mortality in young and middle-aged men [15]. Interestingly, exogenous E2 treatment has also been associated with increased venous thromboembolism risk in transfeminine persons and overall mortality in men with chronic heart failure and reduced left ventricular ejection fraction [16, 17]. These findings suggest that E2 effects depend in part on sex chromosome complement and gonadal type to determine sex-specific differences in disease susceptibility and resistance.

Despite the clinical recognition of these disparities between sexes, the fundamental molecular mechanisms by which E2 orchestrates hepatic responses remain poorly understood at the cell type level. Traditional bulk transcriptomic approaches do not possess the capacity to resolve the cellular heterogeneity of the liver, where parenchymal hepatocytes, together with non-parenchymal populations such as Kupffer cells (KCs), hepatic stellate cells (HSCs), and cholangiocytes, may respond differently to E2. In this study, we used single-nucleus RNA sequencing (snRNA-seq) to elucidate whether and how exogenous E2 differentially regulates gene expression programs between XX and XY genotypes at a single-cell resolution in a gonadectomized and HFD-induced obese mouse model. Our analysis revealed both sex-concordant and sex-discordant cellular and molecular effects of E2 treatment on the liver in obesity, which may underlie differential susceptibility to cardiometabolic disorders such as MASLD and cardiovascular disease.

## Methods

### Animal and dietary treatment

Animal and dietary/E2 treatment-related information were described in our previous study [18]. Briefly, C57Bl/6 J mice of both sexes from Jackson Laboratory were first gonadectomized at 9 weeks of age. After a three-week surgical recovery, all mice were transferred to a 60% kcal High Fat Diet (HFD, Research Diets, D12492) ad-libitum to induce obesity prior to estradiol treatment. Starting at week 33, an intraperitoneal injection of 2 μg of 17β-estradiol-3-benzoate (E2) (Sigma Aldrich #E8515) in sesame oil, or sesame oil alone as vehicle treatment, was given every four days to mice of both sexes through Week 47. Treatment groups were XX + Veh (XXV), XX + E2 (XXE), XY + Veh (XYV), and XY + E2 (XYE) (n = 6–8/group/sex). At the end of the experiment at Week 47, mice were sacrificed. Liver tissues (left lateral lobe) were snap-frozen in liquid nitrogen and then stored at -80 °C for snRNA-seq analysis.

### Tissue dissociation and single-cell preparation

Liver tissue was dissociated, and single nuclei were isolated from 3–4 independent tissues/group/sex using a nuclei isolation kit (Invent Cat# NI-024, Plymouth MN). The 3–4 mice per group selected for snRNA-seq were representative of the broader cohort (n = 6–8/group/sex), with body weights consistent with the cohort mean across key experimental time points, including pre-gonadectomy, post-gonadectomy, and at sacrifice, with no statistical outliers identified among them (Fig. S1A). Briefly, ~ 30 mg frozen liver tissue was thawed, cut, and minced with scissors on ice and transferred to a filter cartridge. Cold buffer A was added, and the tissue was ground for 1–2 min, followed by incubation for 8 min on ice. At the end of incubation, the tissue homogenate was resuspended by inverting the tube a few times and centrifuged at 16,000 × g for 20 s. The filter cartridge was discarded, and the pellet was resuspended by vortexing vigorously for 10 s, and centrifuged at 500 × g for 3 min. The pellet was resuspended in 800 µL cold buffer B and centrifuged at 600 × g for 8 min. The pellet was resuspended in 1 mL of 0.1% BSA/PBS solution. The cell suspension was filtered through a 40 µm Flowmi Cell Strainer (Fisher Scientific, Hampton, NH, USA) to remove nuclei aggregates and debris. Nuclei were centrifuged, and the final nuclei pellet was resuspended in 0.1% BSA/PBS, yielding a final concentration of 700–1200 nuclei/μL.

### snRNA-seq data generation

Single-nucleus RNA-sequencing was conducted following the 10 × 3′ single-cell RNA-seq V3.1 protocol (10 × Genomics, Pleasanton, California, USA). For each group, n = 3–4 independent biological replicates were processed, and a total of 13 independent libraries were constructed. The concentrations of cDNA and library were measured using the Qubit Fluorometric Quantitation method (Thermofisher Scientific, Waltham, MA, USA), and their qualities were evaluated using the Agilent 2200 TapeStation system (Agilent, Santa Clara, CA, USA). The 13 libraries were sequenced on the Novaseq S4 platform with 2 × 100 bp paired-end reads (Illumina, San Diego, CA, USA) in the UCLA Broad Stem Cell Research Center, achieving 25,000 reads/cell. Fastq files were generated by Illumina BCL Convert (Illumina, San Diego, CA, USA). The 10X Genomics CellRanger (v6.1.2) [19] was further used for individual sample demultiplexing, sequencing alignment to *Mus musculus* genome assembly (mm10), barcode and unique molecular identifier (UMI) counting, and feature quantification to generate gene count matrices.

### Ambient RNA decontamination and preprocessing

Output filtered feature-barcode matrix files from CellRanger were processed with CellBender [20] *remove-background* (v0.3.1) to remove ambient RNA contamination. Each sample was run with optimized parameters, including expected-cells, total-droplets-included, fpr, epochs, and learning-rate. Trial runs were initially performed with 20,000 droplets to ensure stability, followed by full runs with adjusted droplet counts to optimize performance. Gene expression counts from CellBender-processed feature-barcode matrices were imported into Seurat (v4.4.0) [21] and merged across all libraries. Cells with fewer than 200 detected genes, more than 2,500 detected genes, greater than 5% mitochondrial transcripts, or more than 5,000 UMIs were removed.

### Cell clustering and cell type annotation

Data were normalized using Seurat’s *NormalizeData*, where feature counts per cell were divided by the total counts, scaled to 10,000, and log-transformed with log1p. Highly variable features (2,000 genes) were selected with *FindVariableFeatures*, and data were scaled with *ScaleData*. Dimensionality reduction was performed with *RunPCA*, retaining the first 30 principal components. A shared nearest-neighbor (SNN) graph was constructed with *FindNeighbors* (k = 25), and clustering was performed with the Louvain algorithm via *FindClusters* across a resolution range of 0.5–4.0. Uniform Manifold Approximation and Projection (UMAP) and t-distributed stochastic neighbor embedding (tSNE) were applied on the top 30 PCs using *RunUMAP* and *RunTSNE* to visualize the cellular landscape in two dimensions. Cluster-specific markers were identified with the Wilcoxon rank sum test through the *FindAllMarkers* function with default parameters. Sample alignment was performed via Canonical Correlation Analysis (CCA), in Seurat. Integration anchors were computed with *FindIntegrationAnchors*, and datasets were integrated with *IntegrateData*. Cell type annotation was performed on integrated clusters at 1.4 resolution. Canonical liver lineage markers from previous liver single-cell/nucleus RNA-seq studies were used to assign broad identities [22–26]. To exclude potential doublets, scDblFinder [27] (v.1.16.0) was used on the raw RNA count matrix. For each cell, a continuous doublet score (scDblFinder.score) and a binary classification (scDblFinder.class = singlet/doublet) were generated. Cells classified as doublets were removed after batch integration and before downstream analyses. Across the dataset, the overall doublet rate was approximately 2.2%, corresponding to the removal of 1,325 cells (2.2% of all nuclei). The number of nuclei retained per sample ranged from 2,249 to 6,932 across all 13 libraries, with a mean of approximately 5,000 nuclei per sample across all four groups, a yield typical of snRNA-seq.

Due to the overwhelming abundance of hepatocyte-derived transcripts, to further minimize the ambient RNA contamination effects and to strengthen the understanding of cell-type-specific effects, hepatocyte-derived signals were removed from NPC populations prior to downstream analyses. Specifically, the top 300 hepatocyte-specific marker genes were removed from NPCs. The hepatocyte markers were derived from two sources: (1) data-driven significant markers (*p.adj* < 0.05) identified from our own dataset using Seurat’s *FindAllMarkers* function and ranked by log2 fold change, and (2) literature-based canonical hepatocyte markers (*Alb*, *Ttr*, *Apoa1*, *Cyp2e1*, and *Cyp3a25*) [28–35].

To assess whether E2 treatment altered the fraction of each cell population in the two sexes, a two-way ANOVA was fit for each cell type with sex, treatment, and their interaction (sex * treatment) as predictors of cell fraction. Post-hoc Tukey testing was performed on interaction terms, and p-values from ANOVA and Tukey post-hoc tests were adjusted across all cell types with the FDR method, with p.adj < 0.05 as the significance cutoff.

### Differential gene expression analysis to identify differentially expressed genes (DEGs)

To evaluate the transcriptional effects of E2 treatment, we performed differential expression analysis across annotated cell populations. For each annotated cell type, transcriptomes of E2-treated cells were compared with vehicle-treated controls in both sexes (XXE vs. XXV; XYE vs. XYV). Wilcoxon rank sum test was performed via FindMarkers in each sex. All genes expressed in at least 10% of cells within either comparison group were considered for testing. P-values were adjusted for multiple testing using the Bonferroni method, and genes with adjusted p-values < 0.05 were considered significant. Each gene was classified as upregulated (avg.log2FC > 0, p.adj < 0.05), downregulated (avg.log2FC < 0, p.adj < 0.05), or non-significant. While Wilcoxon rank-sum testing was retained as the primary differential expression analysis method to be consistent with our previous study of the adipose tissue [36], two complementary analyses were performed to confirm reproducibility. First, a pseudobulk approach was implemented by aggregating raw counts per sample per cell type, followed by differential testing with DESeq2 (v1.42.1) [37] between treatment groups. Analyses were performed separately for XX and XY samples, comparing estradiol-treated samples with vehicle controls. Second, MAST (v1.28.0) [38] was applied via Seurat’s FindMarkers with nCount_RNA included as a latent variable to account for potential differences in sequencing depth. Complete DEG results from all three methods are provided in Table S2. Only DEGs reproduced by at least two methods are reported in the main text, and DEGs reproduced across all three methods are considered highest-confidence findings and prioritized in the Discussion.

To systematically evaluate the overlap and uniqueness of DEGs between XX and XY comparisons within each cell type, UpSet plots were constructed using the ComplexHeatmap [39] R package (v.2.14.0). For each cell type, significant upregulated and downregulated DEGs from both comparisons (XXE vs. XXV; XYE vs. XYV) were organized into lists that served as inputs for DEG set-based intersection analysis. Top DEGs from each category were selected and listed based on the geometric ranking, as described in our previous study [18].

### Stratified rank–rank hypergeometric overlap (RRHO) analysis to assess global differential expression concordance between sexes

To provide a threshold-free, genome-wide assessment of transcriptional concordance between sexes, complementing the UpSet overlap analysis of significant DEGs, a stratified Rank-Rank Hypergeometric Overlap (RRHO) analysis was performed using the RRHO2 [40] R package (v1.0). Differential expression results regardless of significance from both sex-specific comparisons (XXE vs. XXV and XYE vs. XYV) were used as input for each cell type. To generate comparable ranked lists, each gene was assigned a signed significance score calculated as –log₁₀(p-value) × sign(avg log₂ fold change), where positive values indicated upregulation and negative values indicated downregulation. Genes with *p*-values of zero were replaced with one-tenth of the smallest non-zero *p*-value within the same comparison to avoid undefined log transformations.

The two sex-specific gene lists were restricted to their intersection, ensuring that only genes detected in both datasets contributed to the concordance analysis. RRHO matrices were then generated for each cell type using the *RRHO2_initialize* function with log₁₀-transformed scores and labeled according to the corresponding within-sex comparison. The *RRHO2_heatmap* function was subsequently applied to visualize the overlap landscape, where local enrichment scores represent the degree of concordant or discordant regulation between XX and XY E2 responses across the continuous ranking space.

### Pathway enrichment analysis and visualization

To characterize pathway-level changes, we performed enrichment analysis using a hypergeometric test where each DEG set (i.e., up- or down-regulated DEGs in each cell type in each sex) was compared against four mouse-specific pathway resources: Reactome, Hallmark, KEGG, and Gene Ontology (GO) Biological Process databases. The resulting enriched pathways were subsequently compared between sexes to identify concordant (shared) and discordant (sex-unique or opposite) pathway responses to E2. For each pathway, the enrichment score was computed as the ratio of the Gene Ratio (overlap between module DEGs and pathway genes divided by the number of DEGs in the module) to the Background Ratio (pathway gene count present in the background divided by total detected genes in the study). Multiple-testing correction was applied using the Bonferroni method. Significant pathways were selected based on *p.adj* < 0.05 and at least ten overlapping genes and subsequently visualized with the ggplot2 R package (v 3.4.4).

### Transcription factor (TF) enrichment analysis

To identify potential TFs mediating the transcriptional effects of E2, we performed enrichment analysis on cell-type- and sex-specific DEGs using Enrichr [41]. TF-target relations were sourced from the ChEA2022 database [42], which integrates curated data from ChIP-seq, ChIP-chip, and other TF binding site profiling studies. We assessed the significance of overlap between enriched TFs (*p.adj* < 0.05) in XX and XY mice for each cell type using Fisher’s exact test, with the total number of TFs included in the ChEA2022 database as background. To examine whether sex-shared/specific TFs were reflective of direct regulation of DEGs by E2 treatment, we compared the enriched TFs against the direct targets of mouse ERɑ and ERβ from ChEA2022 using Fisher’s exact test, with the total number of genes detected in the study as background.

### Single-cell gene regulatory network (GRN) construction and weighted key driver analysis

To complement TF enrichment analysis that relies on prior-knowledge-based regulatory inference, we constructed data-driven gene regulatory networks (GRNs) to enable the identification of key regulatory genes from global network topology that may not be captured by existing TF-target databases. Cell-type-specific GRNs were constructed using the SCING pipeline [43], where, for each cell type, “supercells” were generated through Leiden clustering to stabilize expression profiles, followed by iterative GRN inference across bootstrap replicates and subsequent consensus merging to retain reproducible regulatory connections while pruning spurious edges. The resulting cell-type-specific GRNs captured directional regulatory interactions and were subsequently used to perform weighted key driver analysis (wKDA) [44] to identify central regulatory genes mediating hepatocyte-zone-specific transcriptional changes. For each cell type, sex- and direction-specific DEGs were mapped onto the corresponding GRN, and enriched key driver (KD) genes (FDR < 0.05) were identified through a χ2-like statistic as implemented in Mergeomics. Lastly, subnetworks centered on the top 5 KDs from each module (sex + regulation direction) in periportal and pericentral hepatocytes were visualized in Cytoscape [45].

### Cell–cell communication analysis

To evaluate how E2 treatment modulates intercellular signaling within the hepatic microenvironment, cell–cell communication analysis was performed via CellChat (v2.1.2) [46]. The CellChatDB.mouse database was applied to define potential ligand-receptor (LR) interactions based on known murine signaling pairs. For each object, overexpressed genes and interactions were identified, and communication probabilities were computed using a tri-mean model. Only interactions supported by at least ten cells per population were retained to ensure robustness of inferred signaling. The resulting communication networks were aggregated to calculate both the total number of interactions and the overall interaction strength for each condition.

To compare E2 effects within each sex, CellChat objects from the same sex were merged (XXV-XXE or XYV-XYE), enabling direct quantification of E2-induced changes in signaling topology in each sex, including the total number and mean strength of inferred interactions, and outgoing and incoming interaction strengths and counts for each cell type. Results were visualized via bar plots displaying interaction counts and weighted strengths for each treatment group within each sex, highlighting signaling activity and receptivity altered by E2 across hepatic populations. Intercellular signaling affected by E2 was evaluated using CellChat’s pathway-level scatter analysis, which compares changes in outgoing and incoming communication strength for each cell type between vehicle and E2-treated conditions within each sex. For each cell type, signaling changes were visualized as scatter plots summarizing both pathway-specific incoming and outgoing changes in signaling activity following E2 treatment. This approach allows for the identification of cell types that preferentially increase signal production, signal reception, or both in response to E2.

### Connecting sex- and cell-type-specific DEGs with human metabolic traits and diseases

To test whether E2-responsive, cell type- and sex-specific transcriptional programs were enriched for common genetic risk of human traits, we performed Marker Set Enrichment Analysis (MSEA) using the Mergeomics pipeline (Version 1.99) [44]. First, summary statistics for 85 GWAS traits and diseases were obtained from the GWAS catalog [47]. For each study, Marker Dependency Filtering (MDF) was applied to reduce redundancy among variants in linkage disequilibrium (LD) by retaining the top 50% of SNP-trait associations and pruning SNPs using LD70 (i.e., LD > 0.7 by correlation coefficient) based on the central European (CEU) population, followed by SNP-to-gene mapping with GTEx v8 liver eQTLs [48]. Cell type-, sex-, and direction-specific DEGs were used as marker sets for enrichment testing.

For each GWAS trait, MSEA was performed to evaluate whether disease-associated SNPs show stronger enrichment with sex- and cell-type-specific DEGs than random chance. DEG sets with FDR < 0.05 were considered significantly enriched. For visualization, we mainly focused on metabolic and cardiometabolic traits (e.g., adiposity, lipid levels, glycemic traits, type 2 diabetes, and coronary artery disease, etc.) with significant enrichment results. Kupffer cells were excluded from the visualization due to the lack of significant enrichment. Enrichment results were summarized as a heatmap of –log_10_(FDR), highlighting significant module-trait associations that reflect potential links between E2-responsive liver programs and human metabolic disease risk.

### Statistical analysis

All statistical analyses were conducted in R (v4.3.2). For comparisons involving sex (XX, XY) and treatment (vehicle, E2), two-way analysis of variance (ANOVA) model was used to test main and interaction effects. Post hoc pairwise comparisons among combined sex × treatment groups were performed using Tukey’s multiple comparison test. Multiple testing correction across cell types was performed using the Benjamini–Hochberg FDR method. Statistical significance was defined as FDR or *p.adj* < 0.05 unless otherwise stated.

## Results

### snRNA-seq identified zonation-specific hepatocytes and non-parenchymal cell types (NPCs)

**Fig. 1:**
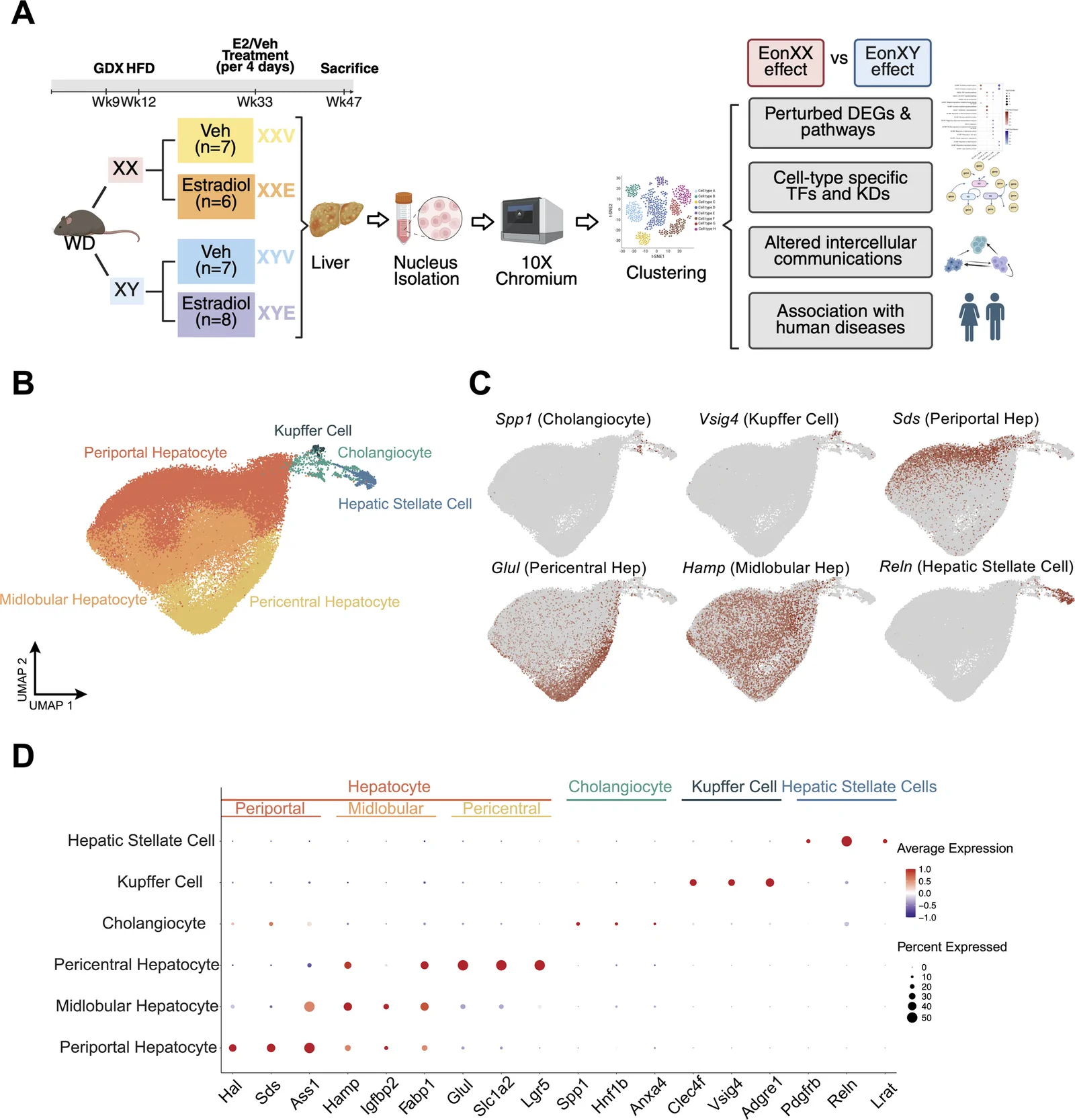
Single-nucleus atlas of liver tissue in diet-induced obese, gonadectomized mice of both sexes. (**A**) Experimental workflow and snRNA-seq analyses of mouse liver. (**B**) Uniform manifold approximation and projection (UMAP) of clusters comprising 58,881 mouse liver nuclei (n = 3–4/sex/treatment; ~ 5000 nuclei/sample). (**C**) Feature plots showing the expression of canonical marker genes for each cell type. (**D**) Dot plots showing the scaled expression of canonical marker genes among cell types

We have previously established a gonadectomized obese mouse cohort where both sexes were treated with E2 (17β-estradiol) or a vehicle and examined relevant cardiometabolic phenotypes (circulating E2 level, body weight, body fat/lean mass percentages, and circulating levels of coagulation factors) [18]. Briefly, C57BL/6 J mice of both sexes (n = 13–15/sex) were first gonadectomized at Week 9 of age, followed by HFD feeding starting at age Week 12 to induce obesity (Fig. 1A). Intraperitoneal injection of E2 (indicated as XXE or XYE) or vehicle treatment (indicated as XXV or XYV) was administered every four days from Week 33 to Week 47. Mice were sacrificed at week 47 for snRNA-seq analyses.

At the phenotypic level, E2 led to trends in lowered weight gain, significant reduction in fat mass percentage (*p* < 0.001 in XX mice and *p* < 0.05 in XY mice), and increased lean mass percentage (*p* < 0.001 in XX mice and *p* < 0.05 in XY mice) in both sexes, with stronger effects observed in XX mice [18]. Circulating levels of coagulation factors VII, VIII, and X did not show significant changes in either sex following E2 treatment [18]. Liver-specific phenotypic measurements were not available for this cohort due to a lack of access to experimental facilities during COVID.

We conducted snRNA-seq on dissected liver tissues (n = 3–4 independent tissues/group/sex) using the 10X Genomics platform (Fig. S1A; details in Methods). After removing low-quality cells (Fig. S1B) and doublets (Fig. S1C), a total of 58,881 high-quality singlets were left for downstream analyses, with an average of ~ 5 k nuclei/sample and a range of 2,249 to 6,932. The number of nuclei is in the expected range for snRNA-seq. A total of 21 cell clusters were identified using Seurat Canonical Correlation Analysis (CCA) [49] and Uniform Manifold Approximation and Projection (UMAP) visualization (Fig. S1D).

By comparing cluster-specific marker genes with cell-type marker genes from previous studies [22–24, 26], we identified three zonation-specific hepatocyte subtypes (periportal-, midlobular-, and pericentral hepatocytes), as well as major NPC types such as KCs, cholangiocytes, and HSCs (Fig. 1B**)**. Canonical marker genes [25] showed cell-type-specific expression as expected **(**Fig. 1C-D; Fig. S1E). No significant differences in cell fractions were found between the E2-treatment group and the vehicle group in either sex as assessed by the two-way ANOVA test (Fig. S1F; detailed statistics in Table S1).

### Sex-shared and discordant effects of E2 treatment on liver cell type transcriptome

**Fig. 2:**
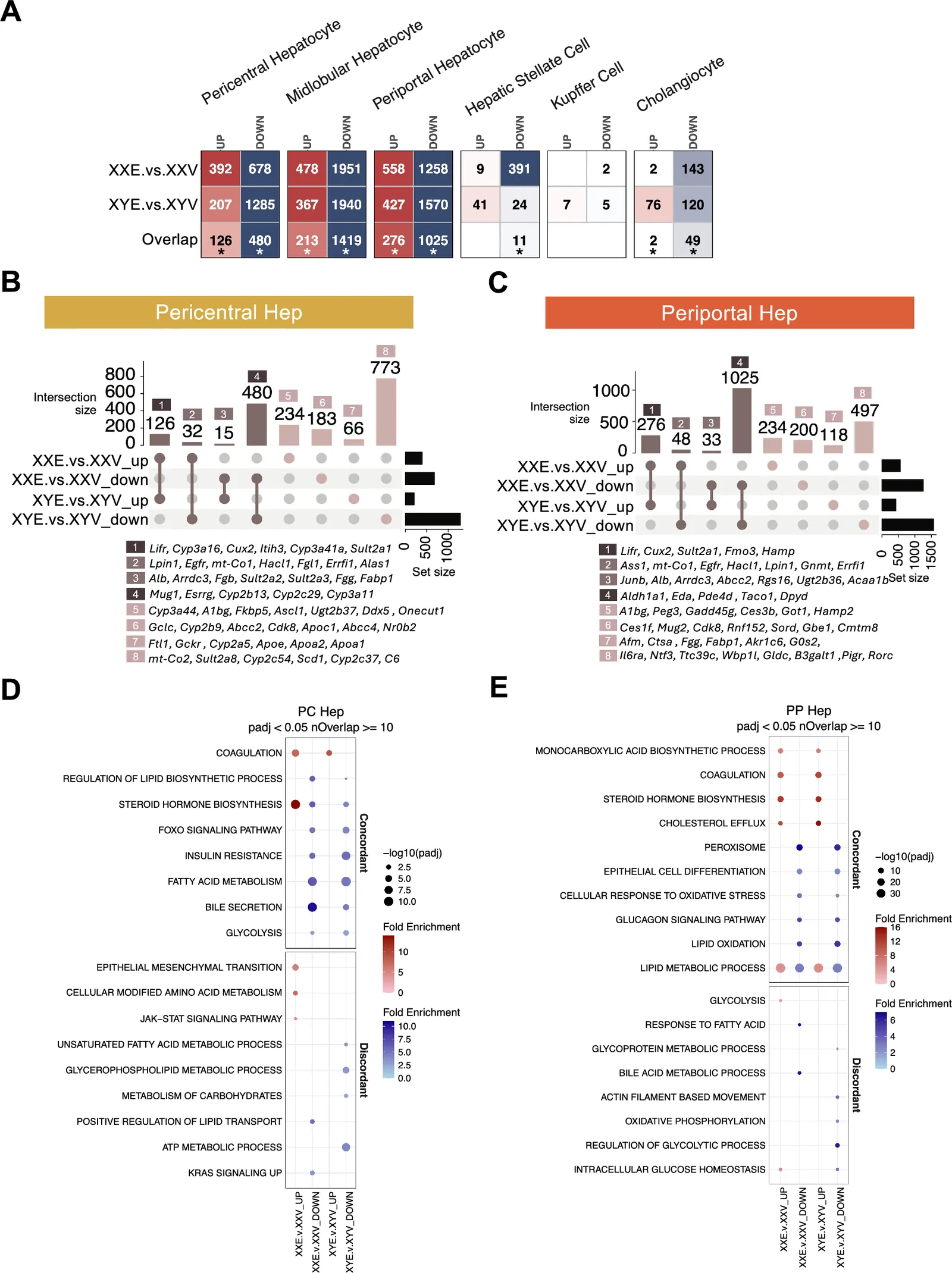
Sex-concordant and discordant transcriptomic changes and altered molecular pathways in response to E2 treatment. (**A**) Number of significant E2-associated differentially expressed genes (DEGs) at *p.adj* < 0.05 in XX and XY mice. (**B**-**C**) Upset plots of DEGs regulated by E2 in pericentral (**B**) and periportal (**C**) hepatocytes, respectively, with top DEGs listed for each category. (**D**-**E**) Representative pathways (*p.adj* < 0.05 and at least 10 DEGs enriched) concordantly and discordantly enriched in pericentral (**D**) and periportal (**E**) hepatocytes in response to E2 treatment. Red dots represent pathways enriched with upregulated DEGs, whereas blue dots represent those enriched with downregulated DEGs. *, *p* < 0.05 by Fisher’s exact test

To examine how E2 treatment affects transcriptomic profiles of individual liver cell types in the two sexes, we conducted differential gene expression analysis using Wilcoxon Rank Sum test as the primary method to identify significant differentially expressed genes (DEGs) at *p.adj* < 0.05 (Table S2), to be consistent with our previous study of the adipose tissue from the same mouse cohort [36]. MAST [38], which adjusts for library size, and sample-level pseudobulk-based differential expression analyses were additionally performed to assess reproducibility (Table S2). We found significant overlaps in the DEGs across methods as assessed by Fisher’s exact test, but the pseudobulk method identified far fewer DEGs consistent with its overly conservative property [50, 51]. We report individual DEGs and pathways that are consistent in at least two methods. Wilcoxon method identified DEGs across cell types in both sexes, with hepatocyte subtypes showing more E2-responsive transcriptional changes than NPC populations. DEG lists showed significant overlap between XX and XY mice in hepatocyte subtypes and cholangiocytes by Fisher’s Exact Test, suggesting shared transcriptional responses to E2 across sexes (Fig. 2A). Given the small cell counts recovered for the NPC populations, these results could likely reflect limited statistical power in the NPCs. We therefore focus on discussing the DEGs from hepatocyte subtypes.

To compare E2 treatment effects between sexes, we used UpSet plots to partition DEGs into sex-concordant E2-associated DEGs (i.e., overlapping DEGs with concordant direction of change between sexes; bar 1 and 4) and discordant DEGs (i.e., unique DEGs to each sex in bar 5–8, or DEGs regulated in the opposite direction between sexes in bar 2 and 3) (Fig. 2B-C, Fig. S2C-F).

In pericentral hepatocytes (Fig. 2B), in E2-treated mice of both sexes, 480 genes were concordantly downregulated (bar 4), and the top genes included *Cyp2b13* and *Esrrg*, which are mainly involved in xenobiotic, lipid, and bile acid metabolism; 126 genes were concordantly upregulated by E2, with *Lifr*, *Cux2*, *Itih3*, *Sult2a1* as top representative genes, whose roles are involved in hepatocyte recovery and regeneration [52], female-biased transcriptional regulation [53], hepatokine protection against steatosis [54], and steroid and bile acid sulfation [55], respectively. Among the discordant DEGs between sexes, in E2-treated XY mice only, 773 genes were uniquely down-regulated (bar 8), including the top genes *mt-Co2*, *Sult2a8*, *Cyp2c54*, *Scd1*, *Cyp2c37*, and *C6* which are mainly involved in oxidative metabolism, bile acid detoxification, unsaturated fatty acid synthesis, as well as complement activity; 66 DEGs were found to be uniquely upregulated in E2-treated XY mice only, including *Cyp2a5*, *Ftl1*, and *Gckr* (Fig. S2A), which are associated with xenobiotic metabolism, iron storage, and regulation of hepatic glucose uptake.

In periportal hepatocytes, 1025 DEGs were concordantly downregulated (bar 4) (Fig. 2C). Examples of shared downregulated DEGs include those associated with fatty acid oxidation and lipid metabolism (*Aldh1a1),* catabolism of pyrimidine nucleotide* (Dpyd),* mitochondrial respiration *(Taco1*), and inflammatory and fibrogenic signaling (*Eda* and *Pde4d).* In E2-treated XY mice only, 497 DEGs were uniquely downregulated (bar 8), including genes involved in IL-6 cytokine responsiveness (*Il6ra*), niche-derived mitogenic signaling (*Ntf3*), and one-carbon and glycosylation metabolism (*Gldc*, *B3galt1*).

In midlobular hepatocytes, in E2-treated mice of both sexes, 1419 downregulated genes were shared (bar 4); in E2-treated XX mice, 494 genes were uniquely downregulated (bar 6); and in E2-treated XY mice only, 471 genes were downregulated (bar 8), suggesting greater sex-concordant effects of E2 on the transcriptome of midlobular hepatocytes (Fig. S2B).

In contrast to hepatocytes, NPCs showed more sex-specific E2-induced DEGs with notably fewer DEGs overall, and specific results for each NPC population are provided in supplementary figures (Fig. S2C-E).

To investigate whether estrogen receptors are expressed among liver cell populations, we examined the expression of *Esr1*, *Esr2*, and *Gper1* (Fig. S2F). *Esr1* was the dominantly expressed receptor and was robustly detected across hepatocyte subtypes, consistent with its established role in regulating liver metabolism and functions [56–58].

To complement the threshold-dependent DEG analysis above, we further performed Rank-Rank Hypergeometric Overlap (RRHO) analysis [28] to capture genome-wide transcriptional concordance between sexes without imposing a significance cutoff. RRHO showed significant directional agreement between sexes across cell types, particularly among downregulated transcripts (Fig. S3A-F), indicating that the sex-concordant response to E2 extends beyond statistically significant DEGs to encompass a broader global transcriptional trend. The greater sex-discordance observed among significant DEGs relative to the global RRHO signal likely reflects the fundamental difference between the two approaches: RRHO evaluates directional agreement across all expressed genes, where the dominant concordant trend prevails, whereas DEG-based analysis resolves the smaller subset of statistically detectable sex-discordant genes insufficient in number to drive a global discordant signal at the transcriptome level.

### Sex-concordant and discordant pathways affected by E2 treatment in liver cell types

To elucidate the representative biological pathways and molecular functions affected by E2 treatment between sexes, we conducted pathway enrichment analysis on the concordant and discordant DEGs (Fig. 2D-E; Table S3). In pericentral hepatocytes, in E2-treated mice of both sexes, E2 concordantly downregulated mRNAs whose products influence insulin resistance, FOXO signaling, bile secretion, lipid biosynthesis regulation, and glycolysis pathways while upregulating ones associated with coagulation pathways (Fig. 2D). Among discordant pathways, in XX mice, epithelial-mesenchymal transition and JAK-STAT signaling pathways were uniquely upregulated, whereas regulation of lipid transport was uniquely dampened. In XY mice, unsaturated fatty acid, glycerophospholipid, and carbohydrate metabolism, as well as ATP metabolic process, were uniquely downregulated.

In periportal hepatocytes, in E2-treated mice of both sexes, the main concordantly upregulated pathways included steroid hormone biosynthesis, cholesterol efflux, monocarboxylic acid biosynthesis, and coagulation (Fig. 2E). Concordantly dampened pathways included peroxisome, oxidative stress response, glucagon signaling, and lipid oxidation. The lipid metabolic process pathway showed enrichment of both up- and down-regulated DEGs in both sexes, indicating a complex role of E2 on lipid metabolism. In E2-treated XY mice only, we observed downregulation of oxidative phosphorylation, actin filament-based movement, and regulation of glycolytic process. In E2-treated XX mice, uniquely enriched pathways included upregulated glycolysis and downregulated response to fatty acid. In addition, intracellular glucose homeostasis was upregulated in XX mice but dampened in XY mice, indicating a potential sex-specific role of E2 in mediating glucose homeostasis and metabolism in periportal hepatocytes.

### E2 responsive genes are enriched for concordant transcription factors (TFs) including sex hormone receptors as well as sex-specific TFs across hepatocyte subtypes

**Fig. 3:**
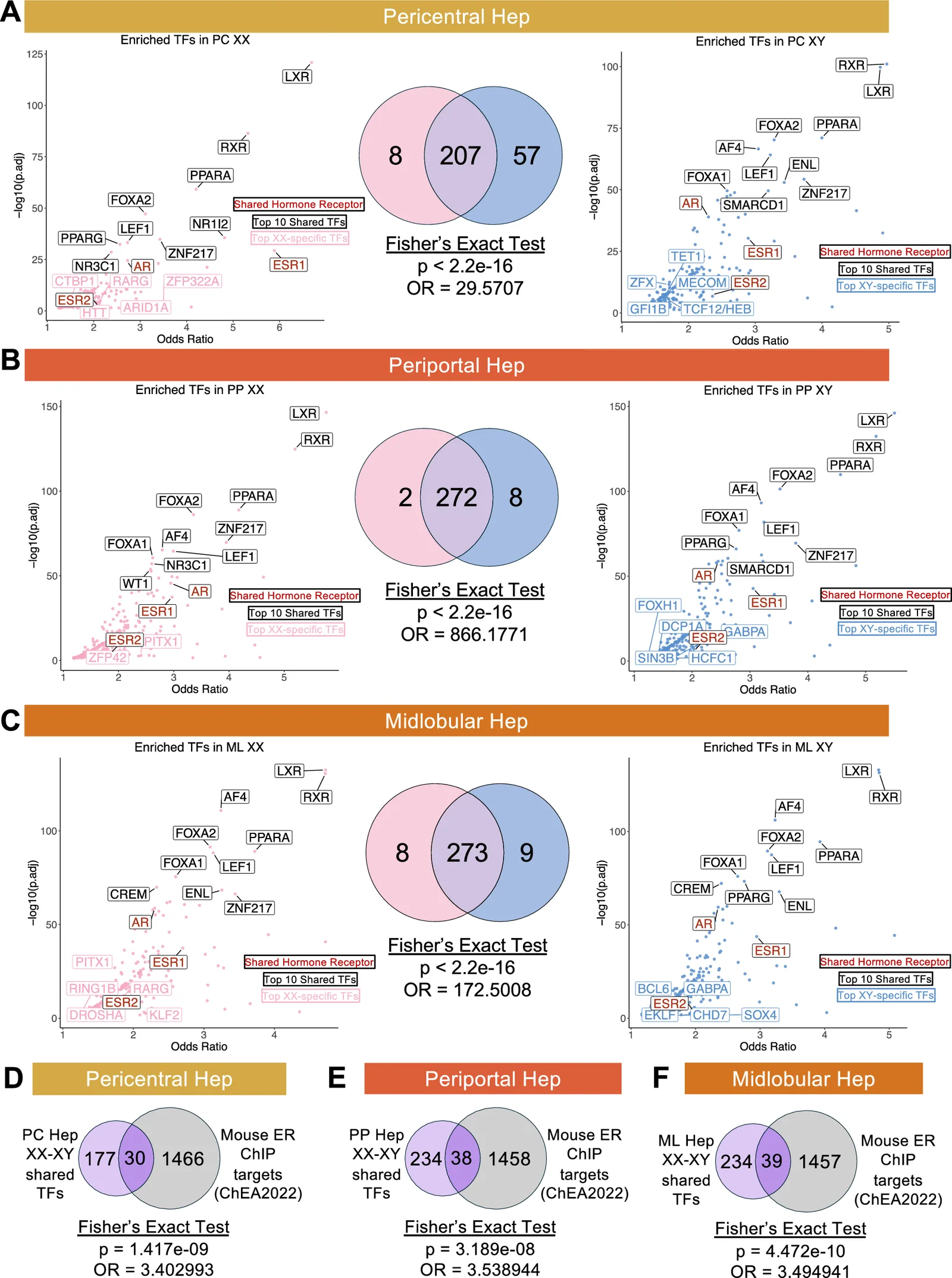
Transcription factor (TF) enrichment analysis on E2-induced DEGs. (**A**-**C**) Significant TFs (*p.adj* < 0.05) enriched in XX (left) and XY (right) mice, respectively, as well as Venn diagram (middle) of overlapping and unique TFs between sexes in pericentral (**A**), periportal (**B**), and midlobular (**C**) hepatocytes. (**D**-**F**) Venn diagram showing overlap between sex-shared TFs in pericentral (**D**), periportal (**E**), and midlobular (**F**) hepatocytes with direct ER targets in mice

To examine the potential regulatory cascades mediating E2 effects, we performed TF enrichment analysis on our sex- and cell-type-specific DEG results using Enrichr [41]. Most enriched TFs were shared between XX and XY mice across all three hepatocyte subtypes (Fig. 3A-C), including sex hormone receptors (ESR1, ESR2, and AR) and core metabolic regulators (LXR, RXR, PPARA, FOXA2, and FOXA1). We also found cell-type and sex-specific TFs. In pericentral hepatocytes (Fig. 3A), eight TFs were XX-specific, including ZFP322A, RARG, CTBP1, HTT, and ARID1A, which are related to the regulation of hepatocyte identity, chromatin remodeling, and metabolic and stress-response [59–61]; 57 TFs were XY-specific, including TET1, ZFX, MECOM, GFI1B, TCF12/HEB, which are mostly associated with tumor growth in the liver [62–66]. In periportal hepatocytes** (**Fig. 3B**)**, the XX-specific TFs ZFP42 and PITX1 are associated with epigenetic regulation of stem cell chromatin states and tumor suppression in the liver [67, 68], respectively; the eight XY-specific TFs, including FOXH1, DCP1A, GABPA, SIN3B, and HCFC1, are involved in developmental signaling, mRNA processing, mitochondrial oxidative metabolism, and epigenetic co-regulation [69–72]. In midlobular hepatocytes (Fig. 3C), XX-specific TFs included RING1B, RARG, DROSHA, KLF2, and PITX1, which are associated with the regulation of hepatocyte proliferation/regeneration and liver metabolic state [73–76]; XY-specific TFs included GABPA, BCL6, and SOX4, which regulate lipid and lipoprotein metabolism as well as stress and injury responses [77–79]. We further looked into how many sex-shared TFs were direct targets of estrogen receptors in the three hepatocyte subtypes. As shown in Fig. 3D-F, around 14% of the sex-shared TFs were direct targets of estrogen receptors in each hepatocyte subtype (significant overlap at p < 0.05 by Fisher’s exact test). Among the sex-specific TFs, overlap with ER targets was generally limited, with 0–1 ER targets among XX- or XY-specific TFs in periportal and midlobular hepatocytes, and 7 out of 57 pericentral XY-specific TFs overlapping with ER targets (Fisher’s exact test, p = 0.016). These findings suggest that a subset of E2-associated transcriptional changes may involve direct ER signaling, particularly among sex-shared TFs, although direct validation (e.g., ChIP-seq) would be needed to confirm this. The other DEGs likely reflect indirect regulatory mechanisms that are further downstream of the adaptive responses to chronic E2 treatment.

### E2 induces conserved and sex-specific gene regulatory networks (GRNs) in hepatocyte subtypes

**Fig. 4:**
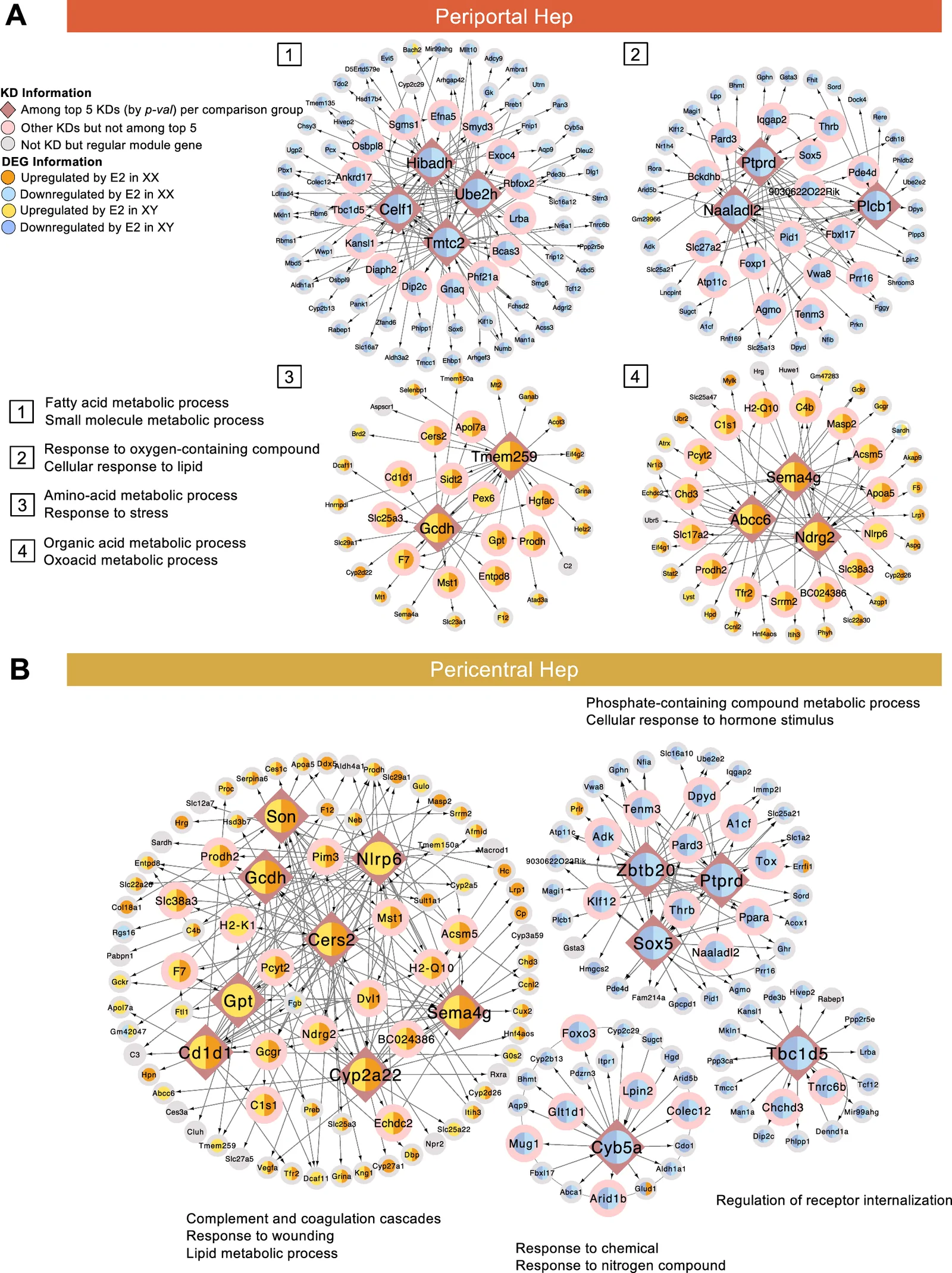
Weighted key driver analysis (wKDA) of E2-induced DEGs in hepatocyte subtypes of XX and XY mice. (**A**-**B**) Gene regulatory subnetworks of top key driver (KD) genes in periportal (**A**) and pericentral (**B**) hepatocytes, with enriched biological functions annotated next to each subnetwork

To complement the prior-knowledge-based TF enrichment analysis, we constructed data-driven GRNs to identify regulatory genes that may not be captured by existing TF-target databases. Using SCING [43], we built cell-type-specific GRNs and applied weighted key driver analysis (wKDA) to identify potential key regulatory genes (key drivers, KDs) mediating E2-induced gene perturbations. In periportal hepatocytes (Fig. 4A), in both sexes, all the top five KDs were concordantly shared. These included *Hibadh*, *Ube2h*, *Celf1*, and *Tmtc2*, whose subnetworks were enriched for downregulated DEGs in fatty acid metabolic process, agreeing with our pathway enrichment analysis (Fig. 2E). Subnetworks of KDs *Ptprd*, *Naaladl2*, and *Plcb1* were enriched for downregulated DEGs involved in response to oxygen-containing compound and cellular response to lipid. Among the top shared KDs for upregulated DEGs, *Gcdh* and *Tmem259* subnetworks were associated with amino-acid metabolic process and response to stress, and *Sema4g*, *Abcc6*, and *Ndrg2* were the top upregulated KDs of subnetworks enriched in organic acid metabolic process and oxoacid metabolic pathways.

For pericentral hepatocyte subnetworks of DEGs (Fig. 4B), the majority (11/13) of their top KDs and associated subnetworks were also regulated in the same direction between the two sexes. One subnetwork containing eight top upregulated KDs had six (*Son*, *Gcdh*, *Cers2*, *Cd1d1*, *Cyp2a22*, and *Sema4g*) shared between sexes and two (*Gpt* and *Nlrp6*) unique to XY. Genes of this subnetwork were enriched in complement and coagulation cascades (including *F7* and *C1s1*), response to wounding (including *Vegfa* and *Mst1*), and lipid metabolic process (including *Gcgr*, *Pcyt2*, and *Apoa5*), consistent with the concordant effects of E2 on pericentral pathways shown in Fig. 2D. Among the downregulated shared KDs between sexes, *Zbtb20*, *Ptprd*, and *Sox5* and their subnetworks were associated with phosphate-containing compound metabolic process and cellular response to hormone stimulus; the subnetworks of KDs *Cyb5a* and *Tbc1d5* were related to response to chemical and nitrogen compounds and regulation of receptor internalization, respectively.

### E2 treatment affected both shared and sex-specific cell–cell communication networks

**Fig. 5:**
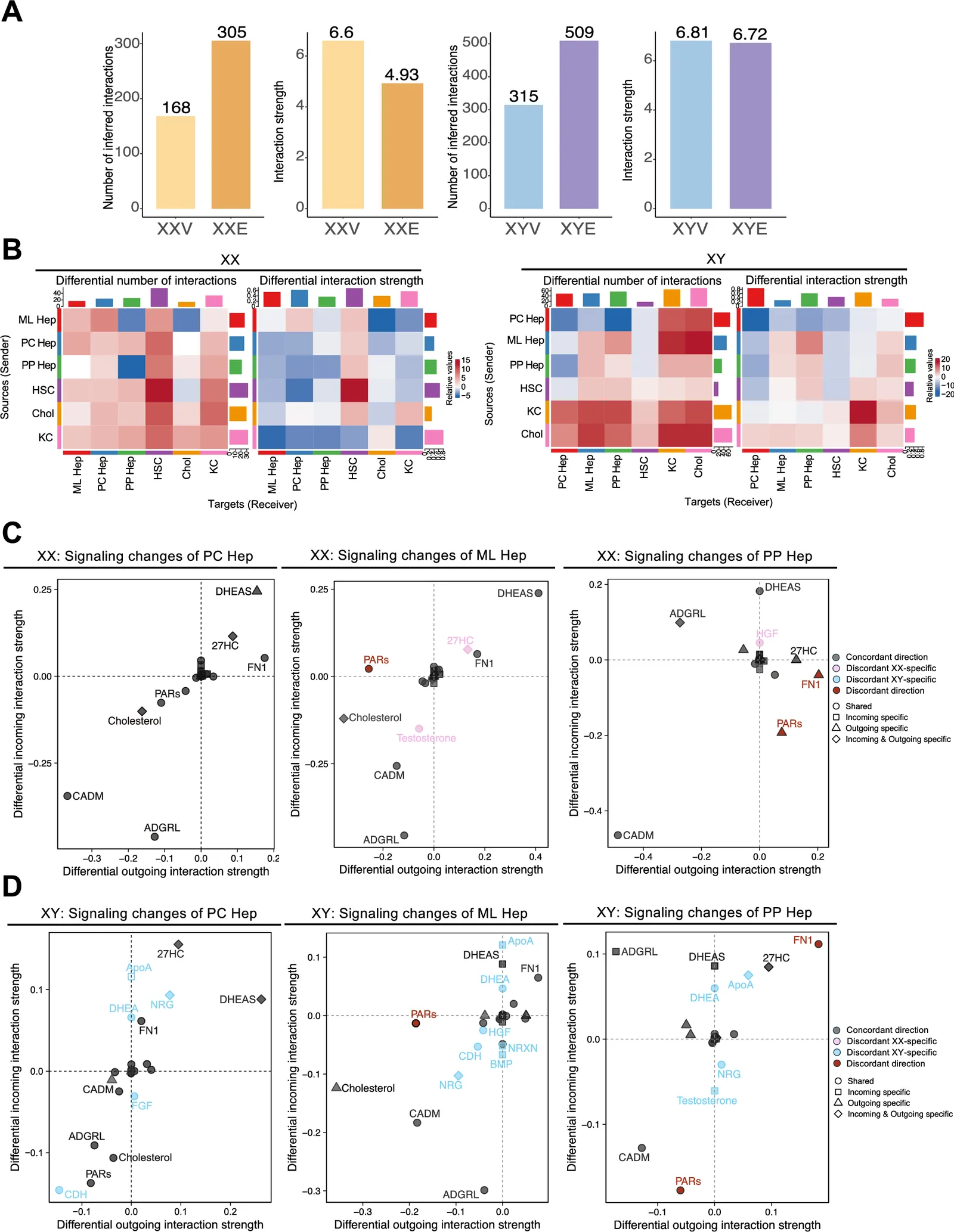
Intercellular communication analyses in response to E2 treatment in XX and XY mice. (**A**) Number of inferred interactions and interaction strength between vehicle and E2 group of each sex. (**B**) Heatmap of interaction number and strength between each pair of cell types in XX (left) and XY (right) mice. (**C**-**D**) Scatter plots of sex-concordant and discordant changes in outgoing (x-axis) and incoming (y-axis) signaling strength across hepatocyte subtypes in XX (**C**) and XY (**D**) mice

In addition to cell type-specific intracellular gene networks affected by E2 treatment, we also investigated how E2 influenced intercellular networks in both sexes. We first examined the changes in the number of interactions and interaction strengths across cell types in the two sexes, respectively (Fig. 5A). In both XX and XY mice, more inferred interactions were observed between cell types in the E2 treatment group. However, the overall interaction strength was reduced (6.6 to 4.93) in XX mice by E2, but remained similar between the vehicle and the E2 groups (6.81 to 6.72) in XY mice. When examining outgoing and incoming interaction numbers and strength (Fig. 5B), we found that in XX mice, E2 induced more incoming and outgoing interactions but dampened the interaction strengths in most cell types, except for HSC which receives more interactions with enhanced strengths from most of the other cell types. In XY mice, E2 induced fewer and weakened incoming and outgoing interactions in pericentral hepatocytes but drove more and enhanced strength of incoming and outgoing interactions in Kupffer cells. Results from NPCs require caution due to the small cell counts.

In terms of sex-concordant and discordant signaling alterations across liver cell populations, we observed overall more dynamic changes in XY mice than in XX mice across hepatocyte subtypes (Fig. 5C-D). In pericentral hepatocytes, E2 treatment resulted in sex-concordant increases in communication strength through steroid precursors and cholesterol metabolism (via DHEAS and 27HC) and FN1 signaling essential for hepatocyte survival post injury [80](Fig. 5C** left; **Fig. 5D** left**). Conversely, both sexes showed reduced communication strength related to cell–cell adhesion (CADM, ADGRL), lipid uptake (cholesterol), and protease-activated receptor signaling (PARs). In addition to shared changes between sexes, XY mice exhibited increased communication strength in signaling related to lipotoxic stress response, autophagy, as well as insulin sensitivity and lipid handling (NRG, DHEA, and ApoA) [81–83], alongside reduced communication through cell adhesion (CDH) and growth factor (FGF) pathways [84, 85]. Similar to pericentral hepatocytes, midlobular hepatocytes also showed shared increases in DHEAS and FN1 communication strength in both sexes (Fig. 5C middle; Fig. 5D middle) as well as concordant reductions in cholesterol, CADM, and ADGRL signaling strength. However, PAR signaling showed discordant patterns, with greater incoming communication strength in XX yet reduced strength in XY. Additionally, XX-specific changes were relatively limited (increased 27HC and reduced testosterone signaling contribution), while XY-specific changes were more extensive, with greater incoming DHEA and ApoA signaling strength and reduced HGF, NRXN, CDH, BMP, and NRG signaling strength. In periportal hepatocytes, both sexes also showed concordant increases in DHEAS and 27HC signaling strength and reduced CADM communication strength (Fig. 5C right; Fig. 5D right). Discordant patterns were prominent: FN1 showed reduced incoming communication strength in XX but increased strengths in XY, and PAR signaling contributed more to outgoing communication in XX but less in XY. E2 also induced XX-specific enhancement of HGF signaling strength, a key growth factor for liver regeneration, and XY-specific increases in ApoA and DHEA signaling strength, along with reduced testosterone and NRG signaling strength, primarily in incoming signals. Among NPCs, consistent with Fig. 5B, XX mice exhibited more signaling alterations in HSCs (Fig. S4A), while XY mice showed greater changes in Kupffer cell communication strength. However, these NPC findings should be interpreted cautiously, given the limited representation of these populations in our dataset.

### Relevance of sex- and cell type-specific E2-induced genes to human metabolic diseases and/or traits

**Fig. 6:**
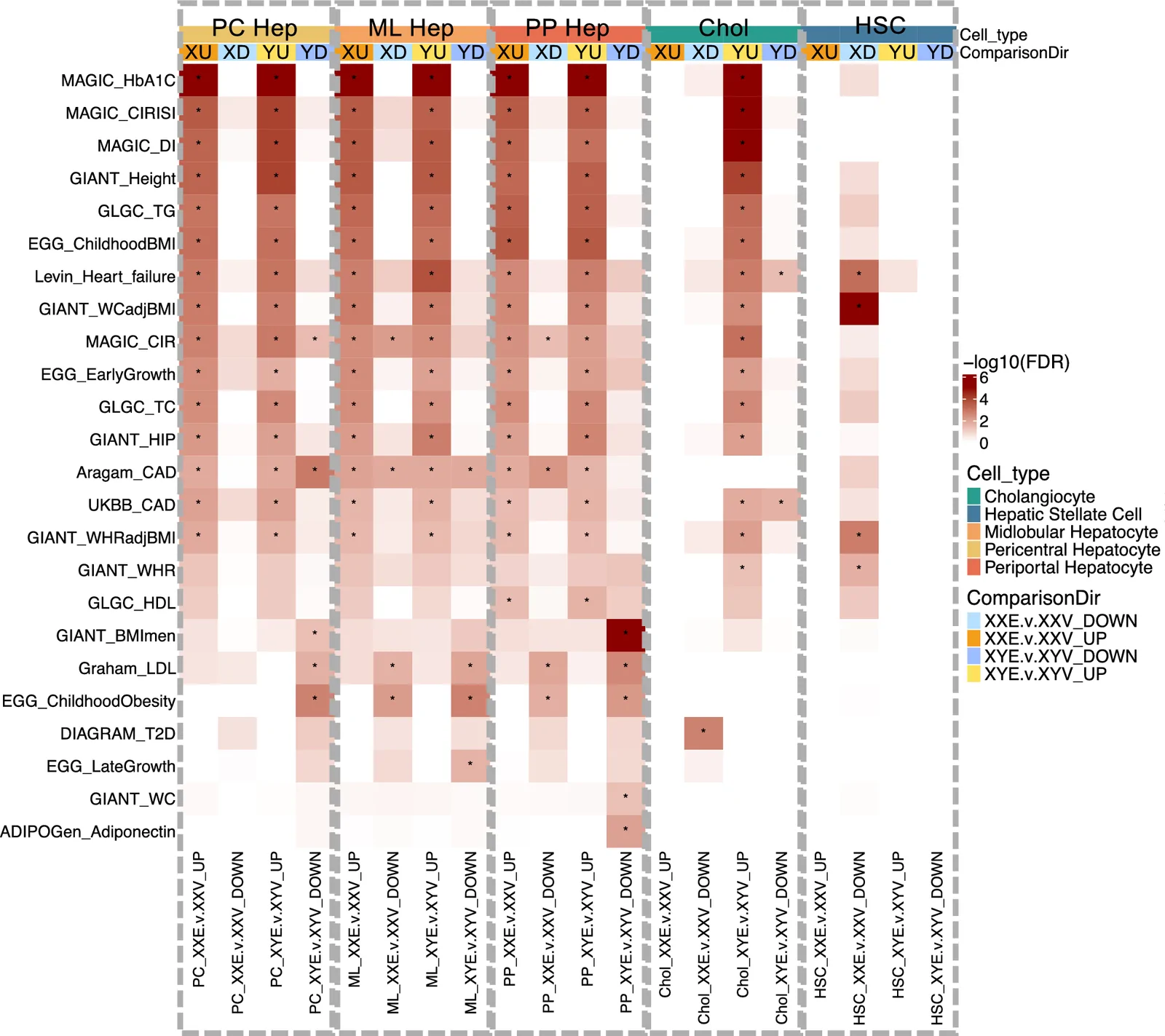
Association of cell-type- and sex-specific E2-associated DEGs with human metabolic disease/trait signals from GWAS studies. Enrichment analysis was performed using Marker Dependency Filtering (MDF) followed by Marker Set Enrichment Analysis (MSEA) in the Mergeomics pipeline. *, FDR < 0.05

Lastly, to determine if genes affected by E2 in liver cell types are associated with human metabolic diseases and traits concordantly or discordantly in the two sexes, we linked our E2-induced DEGs with single nucleotide polymorphisms (SNPs) identified in tens of human cardiometabolic trait/disorder GWAS studies (Fig. 6). Similar trends were observed across the three hepatocyte subtypes, where in mice of both sexes genes upregulated by E2 were enriched for signals associated with triglycerides (TG), heart failure, total cholesterol (TC), and coronary artery disease (CAD). We also noted sex-specific associations, as in E2-treated XY mice only DEGs downregulated in pericentral and periportal hepatocytes showed enrichment for GWAS signals related to men’s body mass index (BMI). Likewise, genes downregulated in both sexes in midlobular and periportal hepatocytes, along with those downregulated in XY only in pericentral hepatocytes, were enriched for genetic signals associated with low-density lipoprotein (LDL) cholesterol level and childhood obesity, revealing both sex- and cell-type-specific effects. In NPC populations, human trait associations were largely sex-biased and exploratory, given the limited cell representation in our dataset; notable enrichments included lipid profile and heart disease signals with XY E2-responsive genes in cholangiocytes, T2D signals with XX downregulated genes in cholangiocytes, and adiposity-related trait signals with XX downregulated genes in HSCs. These findings collectively suggest that both shared and sex-specific effects of E2 across different liver cell types may modulate cardiometabolic disease susceptibility.

## Discussion

In this study, we utilized snRNA-seq to investigate sex-concordant and discordant effects of E2 treatment on cell composition, cell-type-specific genes and pathways, regulatory cascades and networks in liver cell populations of gonadectomized obese mice, and their potential disease relevance. Our results highlighted that chronic E2 administration broadly reprogrammed hepatocyte subtypes in both sexes, with many concordant pathway-level changes alongside notable sex-specific effects among hepatocyte subtypes.

Pericentral hepatocytes are a primary site of glycolysis, lipogenesis, and partial bile acid metabolism, and their dysfunction contributes to lipid accumulation and impaired glucose handling under obesity. E2 elicited concordant suppression of lipogenic, glycolytic, and insulin-resistance-related programs in pericentral hepatocytes of both sexes, resolving the previously established hepatoprotective effects from bulk liver studies [14, 86–89] to a zone-specific, single-cell resolution. Notably, the concordant E2 response in pericentral hepatocytes may carry potential disease relevance, as E2-responsive genes in both sexes converged on human genetic signals for lipid traits and cardiovascular disease. Beyond the shared effects, XY mice exhibited additional suppression of glucose, lipid, and energy metabolic pathways, suggesting a male-specific transcriptional shift away from the obesity-associated lipogenic and glycolytic state. The sex-specific enrichment of men’s BMI signals in XY-downregulated DEGs further points to a sex chromosome-dependent dimension of E2 effects in this zone.

Periportal hepatocytes control oxidative energy metabolism, gluconeogenesis, and inflammatory tone, and are particularly vulnerable to oxidative and fibrogenic insults in obesity. E2 elicited a largely sex-concordant transcriptional response in this hepatic zone as well. The concordant upregulation of cholesterol efflux programs aligns with prior work that hepatocyte ERα promotes cholesterol transport [56] and reduces lipotoxic burden in hepatocytes [90], while concordant suppression of glucagon signaling is consistent with prior findings that E2 suppresses hepatic gluconeogenesis through ERα-mediated mechanisms [88]. Beyond these established effects, our data also reveal shared downregulation of oxidative stress response pathway in both sexes, suggesting that E2 broadly reduces oxidative metabolic burden in periportal hepatocytes, which is particularly relevant given this zone’s vulnerability to oxidative injury under obesity and MASLD [91]. Beyond the concordant effects, XY and XX mice showed divergent regulation of oxidative phosphorylation and glucose-lipid utilization programs, accompanied by sex chromosome-specific enrichment of human disease signals, including waist circumference and adiponectin signals in XY-downregulated periportal DEGs.

Across hepatocytes, network analyses inferred concordant TFs, including ER-related factors and lipid-sensing nuclear receptors, supporting a conserved transcriptional regulatory architecture underlying the shared E2 response. The largely concordant effects of E2 in hepatocytes of both sexes should be interpreted within the broader context of hepatic sexual dimorphism. The liver is among the most sexually dimorphic organs, shaped by a complex interplay of sex hormones, growth hormone signaling, sex chromosome-linked gene regulation, aging, and circadian factors [92]. Our current study only addresses a narrower and more specific question regarding the role of estradiol in liver gene regulation. Our findings indicate more concordant and less discordant responses to E2 in individual liver cell types between male and female mice in the absence of other gonadal hormones, thereby suggesting that estrogen may not be a primary driver of this dimorphism in the liver. Consistent with this notion, our previous work using the Four Core Genotypes (FCG) mouse model [93] demonstrated that testosterone exerts a stronger transcriptional effect on the liver than estradiol. Therefore, the largely sex-concordant E2 effects we observe in hepatocytes reflect the more limited and specific scope of estradiol’s transcriptional influence in the liver, and hepatic sexual dimorphism is likely more attributable to non-estrogen sex-biasing factors.

Comparing E2 effects in liver versus visceral adipose tissue from our previous study [18] reveals strong tissue-specificity: adipose stem and progenitor cells and macrophages showed mostly XX-specific E2 responses, whereas liver exhibited more concordant effects between sexes, particularly in hepatocyte subtypes. These results suggest that sex concordance/discordance of E2 transcriptional effects might be cell-type and tissue-dependent.

Additionally, compared to our prior bulk transcriptomic study of healthy gonadectomized FCG mice [93], E2 elicited a more prominent transcriptional response in the obese liver in the current study, likely reflecting enhanced E2 sensitivity under an obesity-related metabolic state, though differences in E2 dosage, animal age, and methodology preclude direct comparisons. Nevertheless, the consistent enrichment of organic acid metabolism and complement and coagulation cascades across both studies suggests these represent core hepatic E2-regulated programs independent of metabolic context or age.

Notably, while circulating coagulation factors remained unchanged in our mouse cohort [18], pathway analyses revealed upregulated coagulation-related programs in hepatocytes of both sexes. This aligns with the clinical associations between exogenous E2 treatment and elevated venous thromboembolism risk [94, 95] and suggests that post-transcriptional regulation, such as protein translation, stability, or secretion, rather than transcription alone, may govern E2’s influence on coagulation factor availability. This speculation also partially agrees with a prior rat study [96] showing a temporal gap between peak plasma E2 levels and thrombin generation. Accordingly, further studies are needed to delineate the regulatory mechanisms through which E2 influences hepatic coagulation factor production in the hepatocyte subtypes and their systemic availability.

We acknowledge the following limitations of our study. First, liver-specific phenotypic measurements, including histology and hepatic triglyceride content, were not available for this cohort, limiting direct contextualization of the transcriptomic findings within a defined disease stage. However, we note that a recent study from our group [97] using a similar gonadectomized, E2-treated mouse model showed that E2 significantly increased hepatic AKT phosphorylation in both sexes, potentially consistent with our transcriptional finding of concordant suppression of FOXO signaling pathways in hepatocytes of both sexes. The phenotypic findings of both studies are also consistent with regard to E2 effects on body weight and composition. Future studies incorporating comprehensive liver phenotyping alongside transcriptomic profiling will be essential for more definitive interpretation. Second, the 14-week chronic E2 treatment here may reflect long-term transcriptional adaptations rather than acute E2 responses, and shorter treatment durations may yield distinct sex-specific hepatic profiles. Future time course studies are warranted to understand the dynamics of the E2 response. Third, snRNA-seq is prone to ambient RNA contamination from the dominant cell types, in this case, hepatocytes, and indeed, we found high levels of hepatocyte genes across all cell types, including NPCs. Although we applied CellBender with rounds of sample-specific tuning and post-processing to remove dominant hepatocyte markers from NPCs, ambient RNA contamination cannot be eliminated entirely, and residual background noise may exert confounding effects on downstream analyses of NPCs. This could have contributed to the removal of NPC nuclei and explained the limited numbers of NPC populations in our dataset. Additional factors that may affect NPC retrieval include the inherent bias of snRNA-seq toward larger transcript-rich cells [98] and cell-type-specific properties that render certain populations, such as endothelial cells, prone to loss during nuclei isolation [99, 100]. Fourth, while *Esr1* expression and TF enrichment analyses may support ERα as a candidate mediator of E2-induced transcriptional changes, this evidence is supportive but not conclusive, and indirect mechanisms such as E2-induced changes in metabolic fluxes or other systemic mediators cannot be excluded. Fifth, future cell-type- and sex-specific technical (e.g., qPCR) and functional validation (e.g., overexpression or knockdown) is needed to support our sex-concordant and discordant effects of E2 on gene and molecular programs in the liver cell types. Lastly, all findings are based on transcriptomic data. As mRNA abundance does not always correlate with protein expression or functional pathway activity [101], our findings should be considered hypothesis-generating and interpreted with caution, pending future functional validation.

### Perspectives and significance

This work reveals the sex concordant and discordant transcriptional effects of E2 across liver cell types of obese gonadectomized mice, highlighting that hepatocyte subtypes shared largely conserved E2-responsive programs between sexes. The discordance in intercellular signaling across liver cell types suggests that E2 effects on cell–cell communication may differ depending on sex chromosomal background and the hormonal context established by gonadectomy. Collectively, these findings extend the understanding of E2’s transcriptional influence on the liver at single-cell resolution, advance knowledge of how E2 treatment modulates hepatic lipid and glucose metabolism, coagulation programs, and intra/intercellular communication in a sex-specific manner, and provide a foundation for investigating sex chromosome-dependent E2 mechanisms relevant to obesity-associated hepatic metabolic dysfunction.

## Conclusions

Our study showed that chronic E2 administration induced largely concordant transcriptomic responses across hepatocyte subtypes in both sexes with additional sex-specific effects, with ERα signaling as a candidate mediator alongside other core metabolic transcription factors and regulatory mechanisms. E2-responsive hepatocyte genes of both sexes were also enriched for human genetic signals linked to lipid and cardiovascular traits, and for pathways relevant to MASLD, including dampened insulin resistance and lipogenic programs in pericentral hepatocytes and reduced oxidative stress response and glucagon signaling in periportal hepatocytes. Sex-specific E2 responses were also observed, supporting a certain level of divergence in E2 effects in metabolic and anti-inflammatory pathways, networks, and potential disease associations. Together, these findings provide cellular and molecular insights into E2’s hepatoprotective role against cardiometabolic disorders across hepatocyte zones and across sexes.

## Supplementary Information

Supplementary Material 1.

Supplementary Material 2.

Supplementary Material 3.

Supplementary Material 4.

## Data Availability

The datasets generated and/or analyzed during the current study are available in the Single Cell Portal and can be accessed with the study number SCP3426.
