# Supplementary material for: Mapping the hepatic regulatory landscape of estradiol in gonadectomized obese female and male mice at single-nucleus resolution
Source: Biol Sex Differ. 2026 Aug 7;17:176. doi: 10.1186/s13293-026-00962-1 (PMC13732953; doi:10.1186/s13293-026-00962-1)
Supplement: Supplementary file 1 [file 13293_2026_962_MOESM1_ESM.docx]

Supplementary Materials for

#### Mapping the Hepatic Regulatory Landscape of Estradiol in Gonadectomized Obese Female and Male Mice at Single-Nucleus Resolution

Yutian Zhao, *et al.*

Corresponding authors:

Xia Yang ([xyang123@ucla.edu](mailto:xyang123@ucla.edu))

John M. Stafford ([John.Stafford@osumc.edu](mailto:John.Stafford@osumc.edu))

#### Supplementary Figures

**Figure S1. Processing and annotation of single-nucleus RNA-seq data of liver tissue.** (**A**) Bar graphs show body weight (g) at pre-gonadectomy (Pre-GDx), post-gonadectomy (Post-GDx), and sacrifice (Sac) for each experimental group. Red dots represent the 3-4 mice selected for snRNA-seq analysis; black dots represent the remaining mice in the cohort. (**B**) Violin plot of the number of detected UMIs (left), features (middle), and percentages of reads mapped to the mitochondrial genome (right). (**C**) UMAP of singlets and doublets identified by *scDblFinder*. (**D**) UMAP of cells grouped by sample after CCA integration. (**E**) Heatmap of enrichment between the top 100 cell-type-specific marker genes identified in the study and top 100 (if any) marker genes from the reference study. (**F**) Bar plot of cell fractions by group. No significant differences in cell fractions were detected between E2-treated and vehicle groups in either sex (two-way ANOVA with FDR correction; all cell types p.adj > 0.05). Details in Table S1. Data are presented as mean ± SEM. XYV, XY vehicle-treated; XYE, XY E2-treated; XXV, XX vehicle-treated; XXE, XX E2-treated.

**Figure S2. Sex- and E2-dependent transcriptional responses across hepatic cell types and estrogen receptor expression in the liver.** (**A**) Violin plots showing expression of the top upregulated DEGs in pericentral hepatocytes specific to XY mice (XYE vs. XYV). (**B-E**) UpSet plots depicting the overlap of DEGs between XX and XY mice in response to E2 treatment in midlobular hepatocytes (**B**), hepatic stellate cells(**C**), cholangiocytes (**D**), and Kupffer cells(**E**). (**F**) Violin plots showing expression levels of estrogen receptors *Esr1*, *Esr2*, and *Gper1* across hepatic cell types. ****, Bonferroni-corrected *p.adj* < 0.0001.

**Figure S3. Stratified rank-rank hypergeometric overlap (RRHO) test.** (**A-E**) RRHO results between XXE vs. XXV (x-axis) and XYE vs. XYV (y-axis) for each liver cell type. (**A**) Pericentral hepatocytes. (**B**) Midlobular hepatocytes. (**C**) Periportal hepatocytes. (**D**) Kupffer cells. (**E**) Cholangiocytes. (**F**) Hepatic stellate cells.

**Figure S4. Intercellular communication analyses in response to E2 treatment in XX and XY mice.** (**A-B**) Scatter plots of sex-concordant and discordant changes in outgoing (x-axis) and incoming (y-axis) signaling strength in Kupffer cells (**A**) and hepatic stellate cells (**B**) of XX and XY mice.

#### Supplementary Tables

**Table S1. Two-way ANOVA results of cell proportion difference analysis.**

**Table S2. Cell-type-specific differentially expressed genes between E2 and vehicle groups in XX and XY mice.**

**Table S3. Cell-type- and sex-specific pathway enrichment results.**
